# Wound-induced signals regulate root organogenesis in Arabidopsis explants

**DOI:** 10.1186/s12870-022-03524-w

**Published:** 2022-03-22

**Authors:** Seung Yong Shin, Su-Jin Park, Hyun-Soon Kim, Jae-Heung Jeon, Hyo-Jun Lee

**Affiliations:** 1 Plant Systems Engineering Research Center, Korea Research Institute of Bioscience and Biotechnology, Daejeon, 34141 Korea; 2 Department of Functional Genomics, KRIBB School of Bioscience, University of Science and Technology, Daejeon, 34113 Korea; 3 Department of Biosystems and Bioengineering, KRIBB School of Biotechnology, University of Science and Technology, Daejeon, 34113 Korea

**Keywords:** ROS, Calcium ion, Root organogenesis, Auxin, Explants

## Abstract

**Background:**

Reactive oxygen species (ROS) and calcium ions (Ca^2+^) are representative signals of plant wound responses. Wounding triggers cell fate transition in detached plant tissues and induces de novo root organogenesis. While the hormonal regulation of root organogenesis has been widely studied, the role of early wound signals including ROS and Ca^2+^ remains largely unknown.

**Results:**

We identified that ROS and Ca^2+^ are required for de novo root organogenesis, but have different functions in Arabidopsis explants. The inhibition of the ROS and Ca^2+^ signals delayed root development in detached leaves. Examination of the auxin signaling pathways indicated that ROS and Ca^2+^ did not affect auxin biosynthesis and transport in explants. Additionally, the expression of key genes related to auxin signals during root organogenesis was not significantly affected by the inhibition of ROS and Ca^2+^ signals. The addition of auxin partially restored the suppression of root development by the ROS inhibitor; however, auxin supplementation did not affect root organogenesis in Ca^2+^-depleted explants.

**Conclusions:**

Our results indicate that, while both ROS and Ca^2+^ are key molecules, at least in part of the auxin signals acts downstream of ROS signaling, and Ca^2+^ acts downstream of auxin during de novo root organogenesis in leaf explants.

**Supplementary Information:**

The online version contains supplementary material available at 10.1186/s12870-022-03524-w.

## Background

De novo root organogenesis is a type of plant regeneration that forms adventitious roots in detached leaves without exogenous hormone supplementation [1–3]. The efficiency of de novo root organogenesis depends on the biological and metabolic conditions of explants, including their leaf age, carbohydrate content, and endogenous hormone signaling [4, 5]. While leaf explants develop adventitious roots near wound sites without carbohydrate supplementation in the light, root organogenesis does not occur in the dark [4], suggesting that the supplementation of carbohydrate energy sources by photosynthesis is necessary for root organogenesis. In addition to carbohydrates, auxin signaling plays a key role in the development of adventitious roots in explants. Exogenous auxin supplementation allows the reduced rooting rates of leaf explants from auxin-deficient old leaves to recover, suggesting that auxin plays a positive role in root organogenesis [4]. As wounding triggers the development of adventitious roots, wound-induced accumulation of jasmonic acid (JA) is also involved in root organogenesis [5]. JA-mediated expression of the genes encoding ETHYLENE RESPONSE FACTOR 109 (ERF109) and ANTHRANILATE SYNTHASE α1 (ASA1) promotes auxin production to induce de novo root regeneration.

Auxin is a key hormone in de novo root organogenesis [4]. Auxin biosynthesis is activated in explants after leaf detachment by YUCCA (YUC) flavin-containing monooxygenases [1, 6, 7]. The expression of *YUC* genes increases within a few hours after leaf detachment [7]. The YUC-induced auxin is then transported to the cells near the wound sites, causing the cells to transition into root founder cells via the induction of *WUSCHEL-RELATED HOMEOBOX 11* (*WOX11*) and *WOX 12* expression [2, 7, 8]. WOX11 and WOX12 are upstream regulators of LATERAL ORGAN BOUNDARIES DOMAIN (LBD), which promote cell division in root founder cells [1]. WOX11 and WOX12 also upregulate the expression of *WOX5* and *WOX7* by directly binding to their promoters for cell fate transition from the root founder cells to root primordium cells [1, 8].

Reactive oxygen species (ROS) and calcium ions (Ca^2+^) are early signaling molecules involved in plant wound responses [9, 10], which propagate from the wound sites to the systemic tissues for cell-to-cell communication and the accumulation of phytohormones for adaptive responses [10–12]. ROS production after wounding is mainly controlled by the NADPH oxidases RESPIRATORY BURST OXIDASE HOMOLOG D (RBOHD) and RBOHF [12, 13]. The role of ROS in different plant wound responses has been studied using RBOH-deficient mutants. JA signaling activation is a representative wound response; however, the expression of JA-responsive genes is largely suppressed by *rboh* mutations [14]. Extracellular ATP-induced stomatal closure is also defective in *rbohd* mutants [15], which may be linked to wound responses as wounding induces the release of ATP into the extracellular matrix and stomatal closure [16, 17]. Ca^2+^ is also a long-distance signaling molecule involved in plant wound responses [11]. GLUTAMATE RECEPTOR-LIKE (GLR) proteins are ion channels involved in changes in the cytosolic Ca^2+^ concentration after wounding [10]. Wound-induced Ca^2+^ propagation and expression of JA-responsive genes have not been detected in *glr3.3 glr3.6* double mutants, indicating the role of Ca^2+^ in mediating plant wound responses.

In addition to wound responses, ROS and Ca^2+^ are important signaling molecules involved in root growth and development. The suppression of ROS production by mutations in *RBOHD* and *RBOHF* genes results in defects in abscisic acid (ABA)-inhibited primary root growth [18]. During lateral root development, ROS promote cell wall remodeling and activate the formation of lateral root primordia, thereby facilitating lateral root emergence [19]. Additionally, ROS production by RBOHC is necessary for root hair development [20]. It was recently reported that RBOHD-mediated accumulation of ROS in seedlings after hypocotyl cutting controls auxin biosynthesis and improves adventitious root development from wound sites [13], demonstrating the positive role of ROS in root development. Ca^2+^ is related to auxin signaling pathways during root development, and changes in nuclear Ca^2+^ signals by nuclear membrane-located ion channels regulate auxin homeostasis, which affects primary root development [21]. Ca^2+^ also acts as a cofactor of Ca^2+^-DEPENDENT MODULATOR OF ICR1 (CMI1)- INTERACTOR OF CONSTITUTIVELY ACTIVE ROP 1 (ICR1) interactions, which regulate auxin responses during primary root development [22]. Ca^2+^ signals have been reported to be involved in lateral and adventitious root development in rice and cucumber [23, 24], demonstrating that Ca^2+^ is a general upstream regulator of root development in different plant species. While ROS and Ca^2+^ play key roles in root development and wound responses, their roles in de novo root organogenesis in detached leaf explants remain largely unknown.

In this study, we identified that early wound signals of ROS and Ca^2+^ are required for de novo root organogenesis in leaf explants. ROS and Ca^2+^ are not involved in auxin biosynthesis and transport; thus, they do not significantly affect the expression of auxin-responsive genes involved in cell fate transition in explants. Further experiments suggested that ROS are independent of auxin signaling, while Ca^2+^ acts downstream of the auxin signaling pathways during root development. Our results suggest the distinct role of ROS and Ca^2+^ in de novo root organogenesis in leaf explants, which will be beneficial in understanding how early wound signals affect root development in detached tissues.

## Results

### ROS are required for de novo root organogenesis in leaf explants

**Fig. 1:**
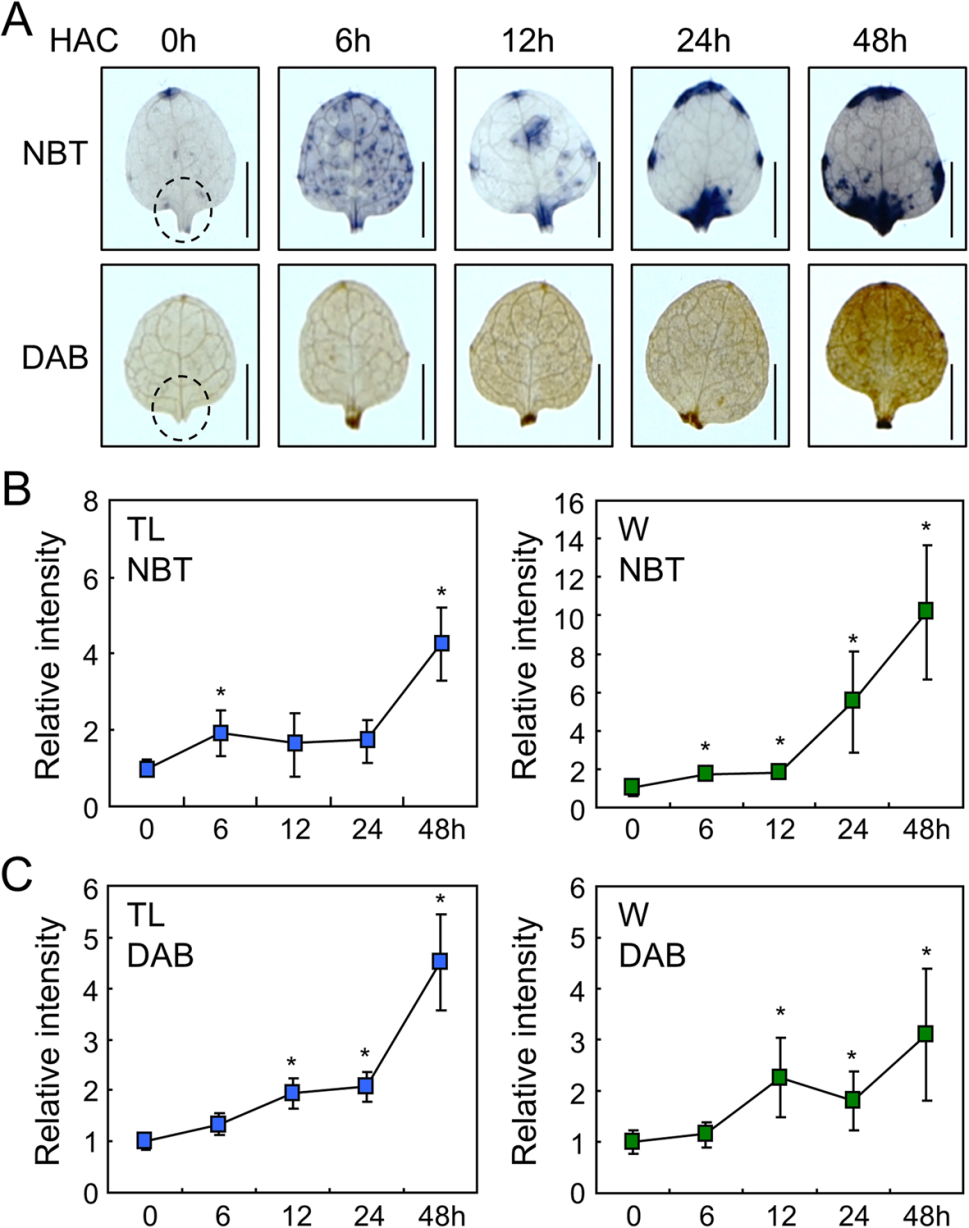
ROS accumulation in the leaf explants. **A** Time course analysis of ROS accumulation in the leaf explants. Leaf explants from the 9-day-old Col-0 seedlings were incubated on B5-agar plates for the indicated time periods before NBT or DAB staining. HAC, hours after culture. Scale bars indicate 0.5 mm. **B-C** Relative intensity of NBT (**B**) and DAB (**C**) signals in the explants. Relative intensity of NBT and DAB signals in the total leaf area (TL) and near the wound site (W) was measured using ImageJ software. Six to nine biological replicates were averaged and statistically analyzed using Student’s *t*-test (*, *P* < 0.05; difference from 0 h). Whiskers indicate ± standard deviations (SD)

In the detached leaves, adventitious roots develop near the wound site without exogenous phytohormone supplementation [4]. As wounding triggers the production of ROS near the wound site [9], we hypothesized that ROS affect root development in leaf explants. To analyze wound-induced ROS in detached leaves, we examined ROS accumulation after leaf detachment using 3,3'-diaminobenzidine (DAB) and nitroblue tetrazolium (NBT), which detect hydrogen peroxide and superoxide, respectively [25, 26]. NBT staining revealed that superoxide accumulated in whole leaves after detachment within 48 h (Fig. 1A, B). The NBT signals near the wound site also continuously increased after leaf detachment (Fig. 1B, right panel). Similar to superoxide, DAB staining showed that the hydrogen peroxide levels gradually increased after leaf detachment throughout the leaf area and in the wound site (Fig. 1A, C). Together, these results suggest that both superoxide and hydrogen peroxide not only rapidly accumulate after leaf detachment near the wound site, but also away from the wound site before root organogenesis.

**Fig. 2:**
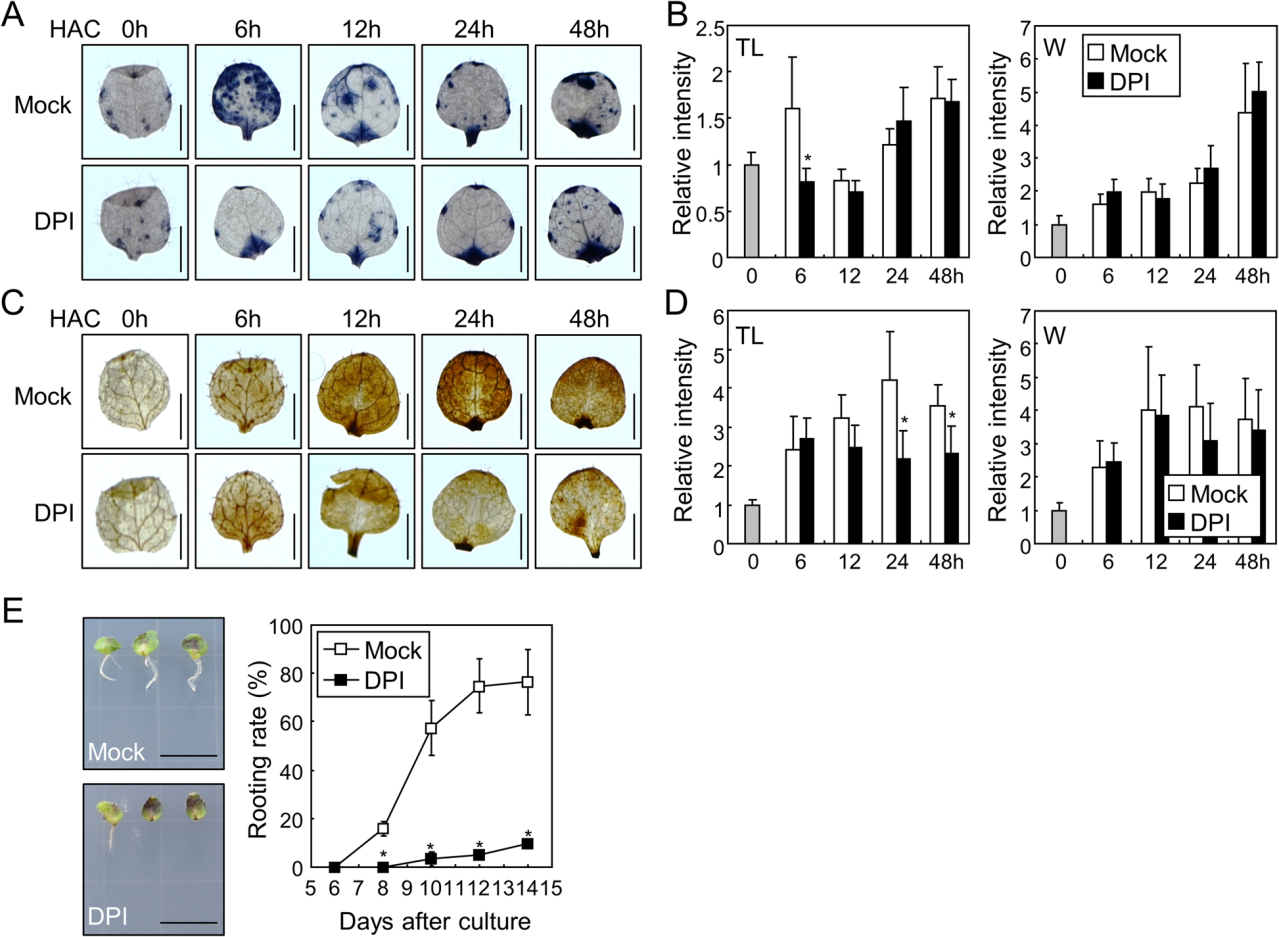
ROS production is required for root organogenesis in the leaf explants. **A-D** Effects of DPI on ROS accumulation in the explants. Leaf explants from the 9-day-old Col-0 seedlings were incubated on B5-agar plates containing 1 µM DPI for the indicated time periods before NBT (**A**) or DAB (**C**) staining. DMSO was used for the Mock control. Relative intensity of NBT (**B**) and DAB (**D**) signals in the total leaf area and near the wound site was measured using ImageJ software. Seven to ten biological replicates were averaged and statistically analyzed using Student’s *t*-test (*, *P* < 0.05; difference from Mock). Scale bars indicate 0.5 mm. Whiskers indicate SD. **E** Effects of DPI on root organogenesis in the leaf explants. Leaf explants from the 9-day-old Col-0 seedlings were incubated on B5-agar plates containing 1 µM DPI up to 14 d. Rooting rates of three biological replicates were averaged and statistically analyzed using Student’s *t*-test (*, *P* < 0.05; difference from Mock). Each replicate contains 20–25 explants. Scale bars indicate 1 cm. Whiskers indicate ± SD

To investigate the role of ROS in de novo root organogenesis, we incubated leaf explants in a phytohormone-free medium containing diphenylene iodonium (DPI), a general inhibitor of ROS-producing RBOH proteins in plants [27]. In NBT and DAB staining experiments, we observed the difference of ROS kinetics in the mock-treated leaves in compared to those in Fig. 1. Possibly because of the highly reactive and unstable nature of ROS, both NBT and DAB signal intensities were highly variable at the specific time point. However, the overall trend in Figs. 1 and 2A-D showed that both superoxide and hydrogen peroxide accumulated in the wound site as well as in the other parts of the leaves within 48 h of detachment. Supplementation with DPI reduced superoxide accumulation at the specific time point after incubation in the total leaf area; however, it did not affect the superoxide levels near the wound site (Fig. 2A, B). Similarly, DPI treatment significantly decreased the accumulation of hydrogen peroxide in leaf explants at some time points in the total leaf area, but did not significantly affect the hydrogen peroxide levels near the wound site (Fig. 2C, D). These results suggest that inhibition of the activity of RBOHs mainly affects ROS production in areas away from the wound site. Next, we measured the rooting rate of leaf explants after DPI supplementation. While the mock-treated explants began to develop adventitious roots at eight days after culture (DAC) and the rooting rate gradually increased up to approximately 80% at 14 DAC, the DPI-treated explants exhibited a rooting rate of approximately 15% until 14 DAC (Fig. 2E, Fig. S1). These data suggest that RBOH-mediated ROS accumulation away from the wound site plays a key role in de novo root organogenesis in leaf explants.

### Crosstalk between RBOH-produced ROS and auxin during de novo root organogenesis in leaf explants

**Fig. 3:**
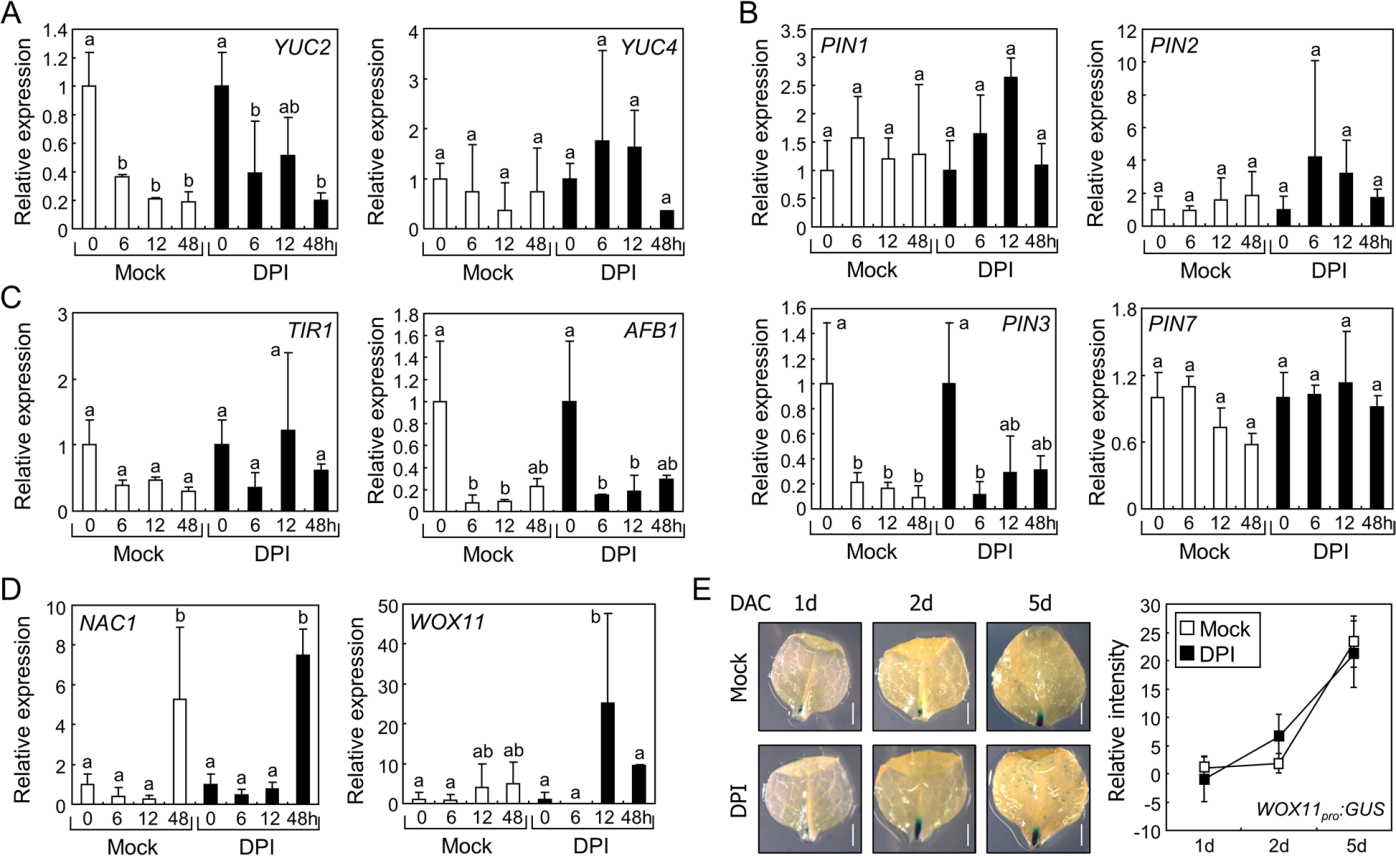
Effects of DPI on gene expressions during root organogenesis. **A-D** Expression of genes involved in root organogenesis. Leaf explants from the 9-day-old Col-0 seedlings were incubated on B5-agar plates containing 1 µM DPI for the indicated time periods. Leaf explants were harvested and expression of genes related to auxin biosynthesis (**A**), auxin transport (**B**), auxin perception (**C**), and root organogenesis (**D**) was analyzed using RT-qPCR. Biological triplicates were averaged. Letters indicate groups that are statistically significantly different from each other (*P* < 0.05, Tukey’s test). Whiskers indicate SD. (**E**) Spatial expression patterns of *WOX11* in the leaf explants. Leaf explants from the 9-day-old *WOX11*_*pro*_*:GUS* transgenic seedlings were used for GUS staining. Leaf explants were incubated on B5-agar plats containing 1 µM DPI for the indicated time periods before GUS staining. Relative intensity of GUS signals near the wound site of the explants was measured using ImageJ software. Nine replicates were averaged and statistically analyzed using Student’s *t*-test (*, *P* < 0.05; difference from Mock). Scale bars indicate 0.5 mm. DAC, days after culture. Whiskers indicate ± SD

Auxin is a critical phytohormone for the development of adventitious roots in explants [4]. As the inhibition of RBOH proteins by DPI treatment largely decreased adventitious root development in leaf explants, we investigated the effects of DPI on the expression of genes related to auxin responses. Auxin biosynthesis by YUC2 and YUC4 in leaf explants is involved in root development [7]; thus, we first analyzed the expression of *YUC2* and *YUC4* genes after DPI treatment. DPI did not significantly change the expression of both *YUC* genes (Fig. 3A), suggesting that RBOH-produced ROS were not involved in regulating *YUC* expression.

In leaf explants, auxin is transported to the wound site to induce root development [7]. As PIN-FORMED (PIN) proteins are the main auxin transporters that determine the directionality of auxin flow in plant cells [28], we analyzed the expression of *PIN* genes in leaf explants during de novo root organogenesis after DPI treatment. The expression patterns of *PIN* genes encoding PIN1, 2, 3, and 7 in mock-treated explants were similar to those in DPI-treated explants (Fig. 3B). Next, we analyzed the expression of genes encoding TRANSPORT INHIBITOR RESPONSE 1 (TIR1) and AUXIN SIGNALING F BOX PROTEIN 1 (AFB1), which are auxin receptors that control adventitious root formation [29, 30]. Again, DPI treatment did not alter the expression of auxin receptor genes (Fig. 3C). To further explore the DPI-mediated suppression of root organogenesis, we analyzed genes involved in cell fate transition. The NAC DOMAIN CONTAINING PROTEIN 1 (NAC1) transcription factor plays a positive role in lateral root development and de novo root organogenesis, but acts independently of auxin during cell fate transition [31, 32]. While *NAC1* expression was significantly induced at 48 h after leaf detachment, DPI treatment did not affect the expression levels (Fig. 3D). We then analyzed the expression of *WOX11*, which is a representative gene involved in auxin-mediated cell fate transition [2]. In our analysis, *WOX11* expression was highly variable in our analysis (Fig. 3D), possibly because *WOX11* is known to be specifically expressed near the wound site [2, 7, 32]. Therefore, we performed β-glucuronidase (GUS) staining for analyzing precise *WOX11* expression using *WOX11*_*pro*_*:GUS* transgenic plants. Time-course analysis of GUS signals indicated that local *WOX11* expression increased after leaf detachment, but was not affected by DPI (Fig. 3E). These results suggest that DPI does not affect the expression of key genes involved in de novo root organogenesis.

**Fig. 4:**
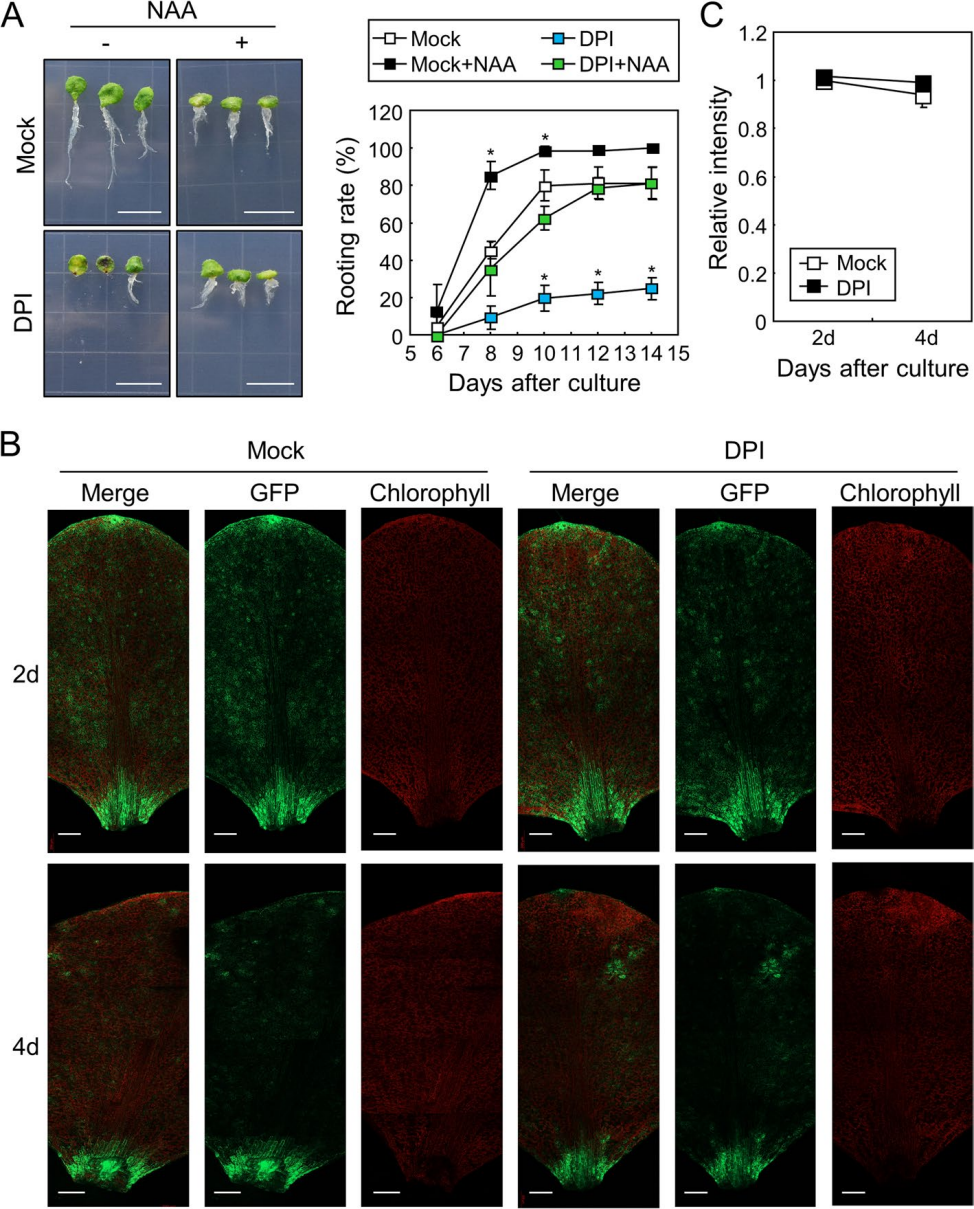
Auxin and ROS independently induce root organogenesis in the leaf explants. Whiskers indicate ± SD. **A** Effects of auxin on root organogenesis in DPI-treated leaf explants. Leaf explants from 9-day-old Col-0 seedlings were incubated on B5-agar plates containing 1 µM DPI with or without 0.1 µM NAA. Biological triplicates were averaged and statistically analyzed using Student’s *t*-test (*, *P* < 0.05; difference from Mock). Scale bars indicate 1 cm. Each replicate contains 20–26 explants. **B-C** Auxin responses in the leaf explants after DPI treatment. Leaf explants from the 9-day-old *DR5*_*rev*_*:GFP* transgenic seedlings were incubated on B5-agar plates containing 1 µM DPI for the indicated time periods. GFP fluorescence and chlorophyll autofluorescence were analyzed using confocal microscopy (**B**). Scale bars indicate 0.2 mm. Relative intensity of GFP signals near the wound site of the explants was measured using ImageJ software (**C**). Four biological replicates were averaged and statistically analyzed using Student’s *t*-test (*, *P* < 0.05; difference from Mock)

To further investigate the relationship between auxin and ROS, we supplemented the mock- and DPI-treated explants with synthetic auxin, naphthalene-1-acetic acid (NAA). As reported previously, NAA treatment elevated the rooting rate by up to ~ 100% in mock-treated explants [4]. Notably, NAA also increased the rooting rate of DPI-treated explants; however, the rooting rate was still lower than that of NAA-treated mock explants (Fig. 4A). We next analyzed the auxin responses by imaging green fluorescent protein (GFP) signals of leaf explants during root organogenesis using seedlings expressing the *DR5*_*rev*_*:GFP* reporter, which is broadly used to study plant auxin responses [33]. GFP fluorescence imaging showed that the GFP signals were notably detectable near the wound site after incubation (Fig. 4B, Fig. S2), but those were not affected by DPI treatment (Fig. 4B, C). Together, these results indicate that at least part of the auxin signals acts downstream of ROS signals, but ROS do not affect auxin responses during de novo root organogenesis in leaf explants.

### Role of Ca^2+^ in de novo root organogenesis

**Fig. 5:**
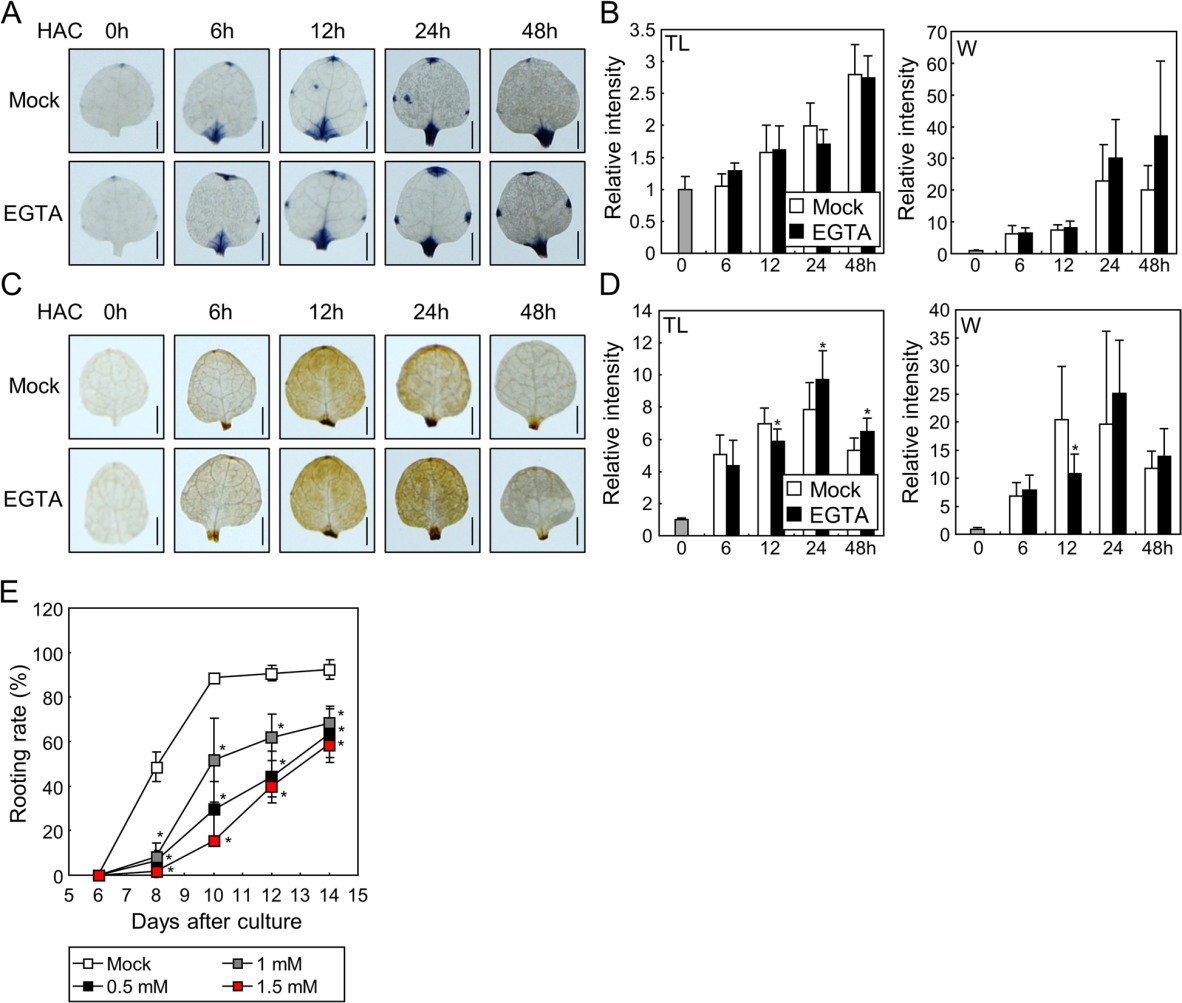
Ca^2+^ signals are required for root organogenesis in the leaf explants. (**A-D**) Effects of EGTA on ROS accumulation. Leaf explants from the 9-day-old Col-0 seedlings were incubated on B5-agar plates containing 0.5 mM EGTA for the indicated time periods before NBT (**A**) or DAB (**C**) staining. Relative intensity of NBT (**B**) and DAB (**D**) signals in the total leaf area and near the wound site was measured using ImageJ software. Seven to ten biological replicates were averaged and statistically analyzed using Student’s *t*-test (*, *P* < 0.05; difference from Mock). Scale bars indicate 0.5 mm. Whiskers indicate SD. (**E**) Effects of EGTA on root organogenesis in the leaf explants. Leaf explants from the 9-day-old Col-0 seedlings were incubated on B5-agar plates containing 0, 0.5, 1, or 1.5 mM EGTA for up to 14 d. Rooting rates of three biological replicates were averaged and statistically analyzed using Student’s *t*-test (*, *P* < 0.05; difference from Mock). Each replicate contains 20–30 explants. Whiskers indicate ± SD

Ca^2+^ is another early signal of plant wound responses. Ca^2+^ signals rapidly increase after wounding and are transmitted to systemic tissues to induce JA responses [10]. Additionally, Ca^2+^ assists ROS production via the activation of RBOH proteins through the function of Ca^2+^-binding proteins and ion channels [20, 34]. Therefore, we hypothesized that Ca^2+^ is also involved in de novo root organogenesis in detached leaves. First, we analyzed the relationship between Ca^2+^ and ROS production in leaf explants by NBT and DAB staining. Again in these experiments, the ROS kinetics in mock-treated leaves were different in compared to those in Figs. 1 and 2A-D, but the overall trend was similar: ROS accumulated both in the total leaf area and in the wound site within 48 h after explant incubation. NBT staining indicated that the suppression of Ca^2+^ signals by the Ca^2+^ chelator, ethylene glycol tetraacetic acid (EGTA), did not affect superoxide accumulation (Fig. 5A, B). DAB staining indicated that EGTA treatment changed the hydrogen peroxide levels in leaf explants at several time points in both areas near and away from the wound site (Fig. 5C, D). To examine the role of Ca^2+^ in root development, we analyzed the rooting rate of detached leaves in phytohormone-free media containing different concentrations of EGTA. A does-dependent decrease in the rooting rate was observed with EGTA treatment (Fig. 5E), suggesting that Ca^2+^ is a positive regulator of root development. Together, these results indicate that Ca^2+^ affects the ROS levels and is required for de novo root organogenesis in leaf explants.

### Relationship between Ca^2+^ and auxin signals during de novo root organogenesis.

**Fig. 6:**
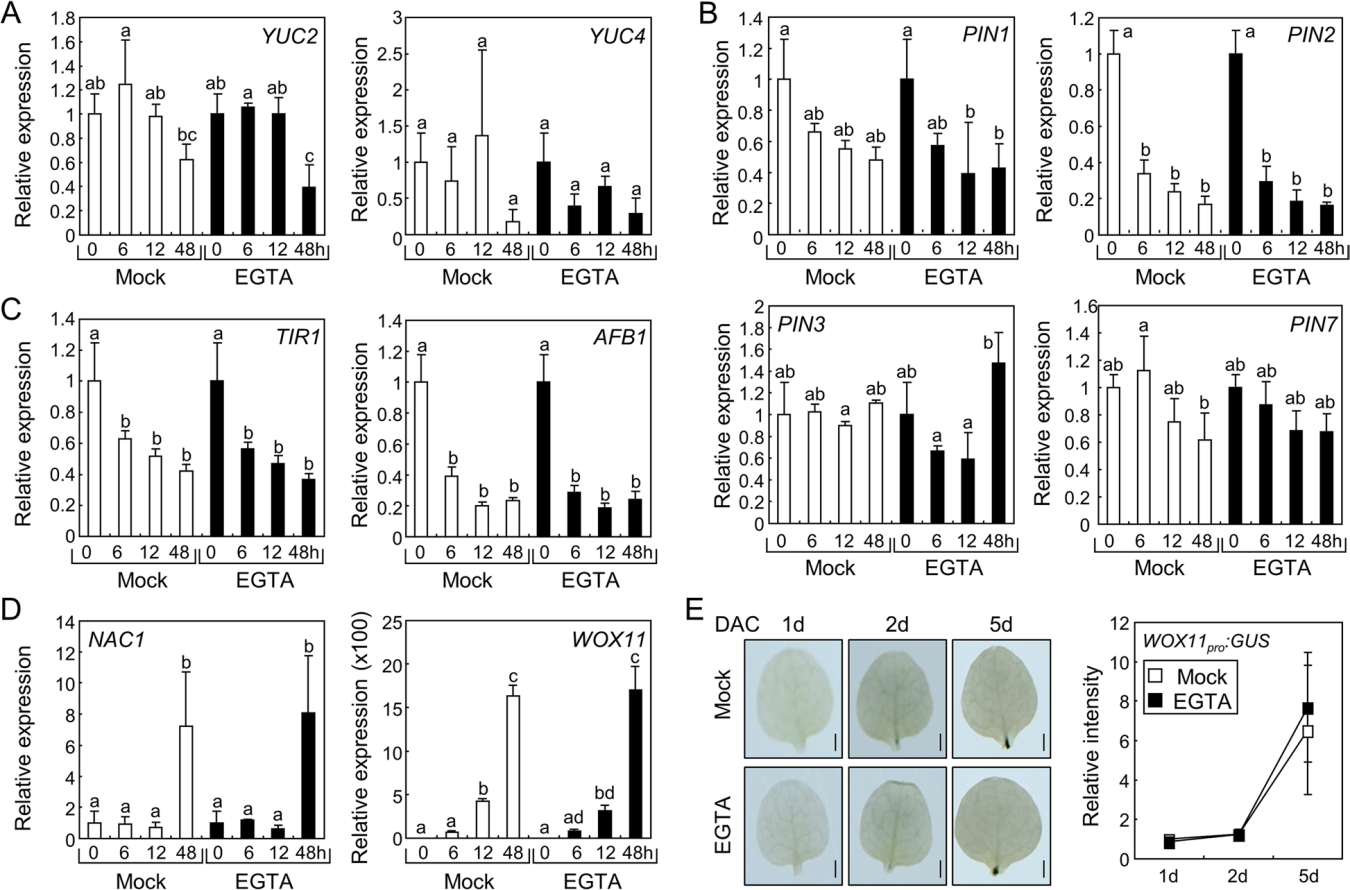
Effects of EGTA on gene expressions during root organogenesis. (**A-D**) Expression of genes related to root organogenesis. Leaf explants from the 9-day-old Col-0 seedlings were incubated on B5-agar plates containing 0.5 mM EGTA for the indicated time periods. Leaf explants were harvested and expression of genes related to auxin biosynthesis (**A**), auxin transport (**B**), auxin perception (**C**), and root organogenesis (**D**) was analyzed using RT-qPCR. Biological triplicates were averaged. Letters indicate groups that are statistically significantly different from each other (*P* < 0.05, Tukey’s test). Whiskers indicate SD. (**E**) Spatial expression patterns of *WOX11* in the leaf explants. Leaf explants from the 9-day-old *WOX11*_*pro*_*:GUS* transgenic seedlings were incubated on B5-agar plates containing 0.5 mM EGTA for the indicated time periods. Relative intensity of GUS signals near the wound site was measured using ImageJ software. Nine biological replicates were averaged and statistically analyzed using Student’s *t*-test (*, *P* < 0.05; difference from Mock). Scale bars indicate 0.5 mm. Whiskers indicate ± SD

To investigate the molecular mechanisms of Ca^2+^-assisted root organogenesis, we analyzed the expression of the gene sets used in Fig. 3. The expression of *YUC*s (Fig. 6A), *PIN*s (Fig. 6B), and auxin receptor genes (Fig. 6C) were not affected by EGTA treatment. Additionally, *NAC1* and *WOX11* genes exhibited similar expression levels in the mock- and EGTA-treated leaf explants (Fig. 6D). Consistent with the reverse transcription-quantitative polymerase chain reaction (RT-qPCR) analysis, the GUS signals in leaf explants from seedlings expressing the *WOX11*_*pro*_*:GUS* reporter were not altered by EGTA treatment (Fig. 6E), indicating that Ca^2+^ is not involved in the expression of key genes in root organogenesis.

**Fig. 7:**
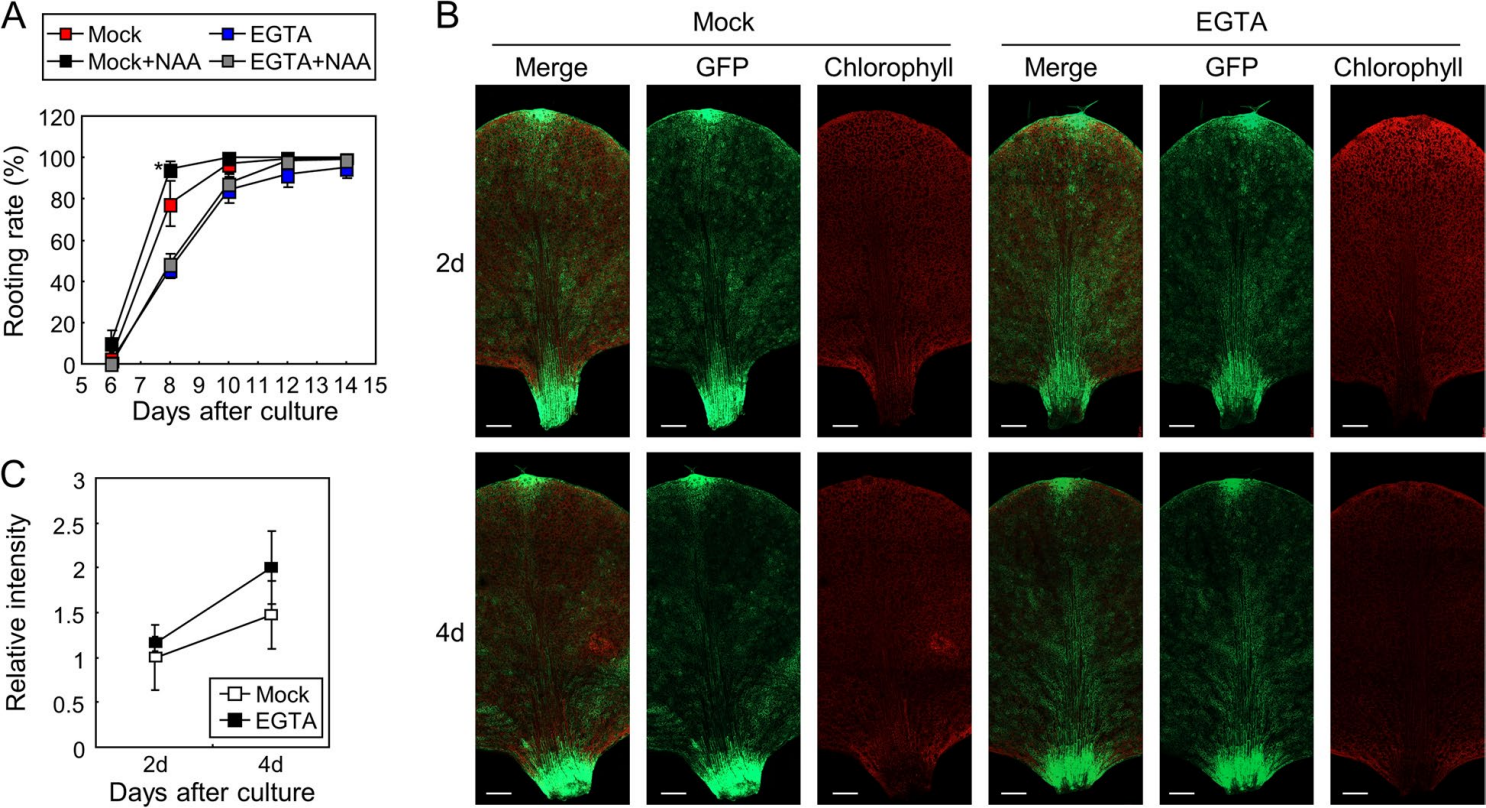
Auxin-induced root organogenesis in the leaf explants is dependent on Ca^2+^ signals. **A** Effects of auxin on root organogenesis in EGTA-treated leaf explants. Leaf explants from the 9-day-old Col-0 seedlings were incubated on B5-agar plates containing 0.5 mM EGTA with or without 0.1 µM NAA. Biological triplicates were averaged and statistically analyzed using Student’s *t*-test (*, *P* < 0.05; difference from Mock). Whiskers indicate ± SD. Each replicate contains 24–30 explants. **B-C** Auxin responses in the leaf explants after EGTA treatment. Leaf explants from the 9-day-old *DR5*_*rev*_*:GFP* transgenic seedlings were incubated on B5-agar plates containing 0.5 mM EGTA for the indicated time periods. GFP fluorescence and chlorophyll autofluorescence were analyzed using confocal microscopy (**B**). Scale bars indicate 0.2 mm. Relative intensity of GFP fluorescence near the wound site was measured using ImageJ software (**C**). Four biological replicates were averaged and statistically analyzed using Student’s *t*-test (*, *P* < 0.05; difference from Mock). Whiskers indicate ± SD

Next, we analyzed the effects of NAA on root organogenesis in EGTA-treated leaf explants. The addition of NAA significantly enhanced the rooting rate in mock-treated explants, but did not change the rooting rate in EGTA-treated explants (Fig. 7A). These results suggest that EGTA blocks auxin signaling during root organogenesis. Thus, we examined the effects of EGTA on auxin responses using *DR5*_*rev*_*:GFP* transgenic seedlings. The fluorescence intensity of GFP was not altered by EGTA treatment (Fig. 7B, C), suggesting that Ca^2+^ acts downstream of auxin signaling to induce root development in detached leaves.

### Analysis of mutants defective in ROS production and Ca^2+^ signaling.

**Fig. 8:**
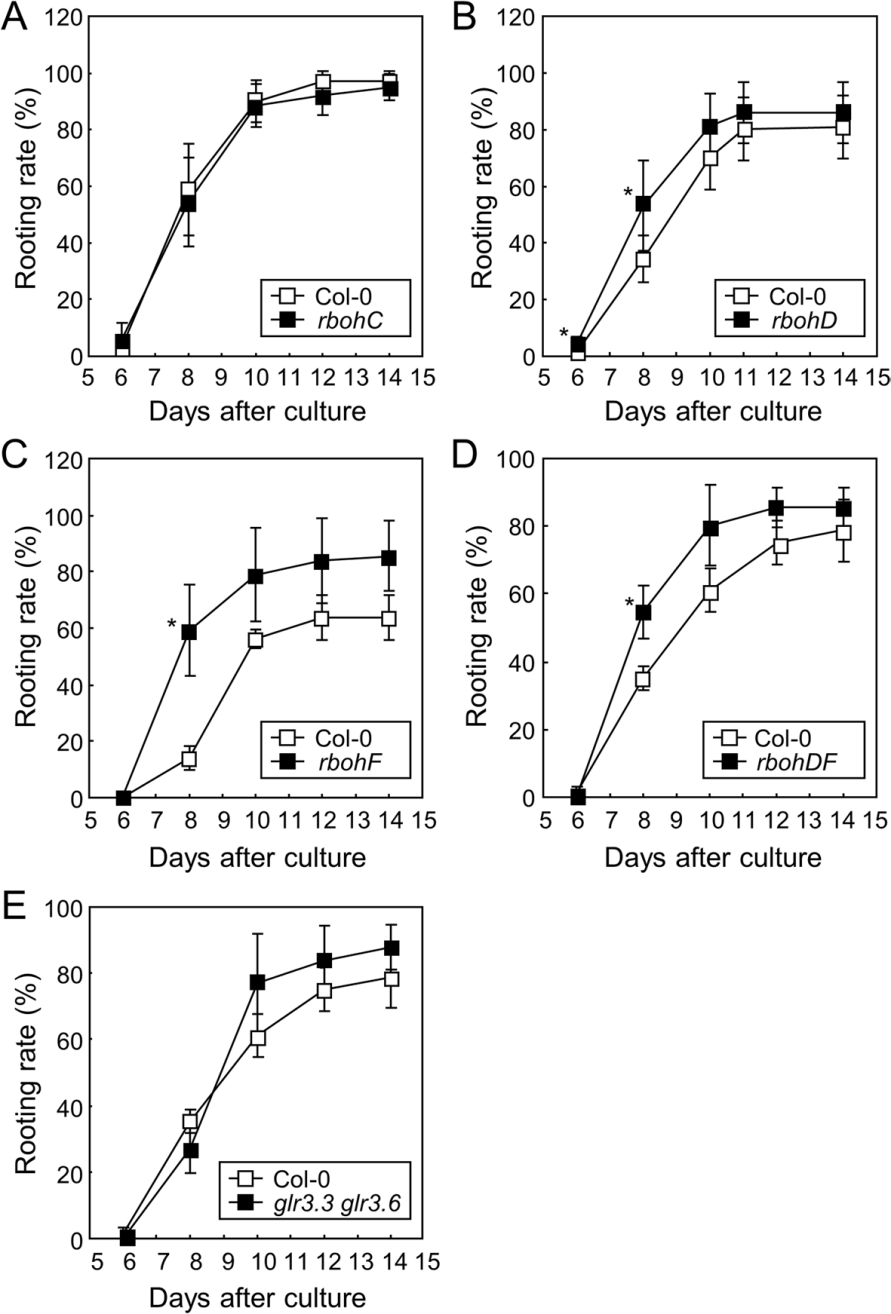
Root organogenesis in the mutants defective in ROS production and Ca^2+^ signaling Leaf explants from the 9-day-old Col-0, *rbohC* (**A**), *rbohD* (**B**), *rbohF* (**C**), *rbohDF* (**D**), and *glr3.3 glr3.6* (**E**) seedlings were incubated on B5-agar plates for up to 14 d. Biological triplicates were averaged and statistically analyzed using Student’s *t*-test (*, *P* < 0.05; difference from Col-0). Whiskers indicate ± SD. Each replicate contains 15–25 explants

Based on our results, the inhibition of RBOHs by DPI largely suppressed root development in leaf explants (Fig. 2E). Among the Arabidopsis *RBOH* family genes, it has been previously reported that *RBOHC*, *RBOHD*, and *RBOHF* are involved in lateral root growth and root hair development [19, 35, 36]. To investigate the role of specific RBOH genes in de novo root organogenesis, we measured the rooting rate of leaf explants from *rbohC*, *rbohD*, *rbohF*, and *rbohDF* mutants. While the *rbohC* mutant and Col-0 exhibited similar rooting rates (Fig. 8A), *rbohD*, *rbohF*, and *rbohDF* mutants exhibited significantly higher rooting rates than that of Col-0 (Fig. 8B to D). These results are inconsistent with our data using DPI, suggesting that the mutation of specific *RBOH* genes is beneficial for root development, while the inhibition of multiple RBOH activities by DPI treatment negatively affects root organogenesis in leaf explants.

To examine the genes involved in Ca^2+^-mediated root development, we used *glr3.3 glr3.6* double mutants, which are defective in wound-induced Ca^2+^ propagation [10]. However, the rooting rate was not affected by *glr3.3 glr3.6* mutations (Fig. 8E), suggesting that GLR-mediated Ca^2+^ propagation is not involved in de novo root organogenesis in leaf explants.

As RBOH-mediated ROS production is affected by Ca^2+^ signals [20, 34], we analyzed whether Ca^2+^-assisted root organogenesis depends on RBOH activity. Leaf explants from Col-0, *rbohC*, and *rbohDF* seedlings were incubated on phytohormone-free media with or without EGTA. The rooting rate measurement indicated that EGTA suppressed root development in both Col-0 and *rboh* mutants (Fig. S3), suggesting that Ca^2+^-mediated root organogenesis does not depend on RBOH functioning.

## Discussion

In this study, we demonstrated that ROS and Ca^2+^ are required for de novo root organogenesis in leaf explants. ROS accumulated in leaf explants after detachment. As the inhibition of RBOHs using DPI largely decreased root development (Fig. 2E), RBOH-mediated ROS production is necessary for root organogenesis in leaf explants. Our data showed that DPI treatment did not affect auxin responses (Fig. 4B) and that auxin treatment partially restored the rooting rate suppressed by DPI (Fig. 4A). These results suggest that ROS and auxin signals have some crosstalk, but ROS do not affect auxin responses at least within our observed time periods during de novo root organogenesis.

The inhibition of Ca^2+^ signals by EGTA treatment resulted in different results. While EGTA and NPA treatment similarly inhibited root development in leaf explants, auxin treatment completely restored the EGTA-mediated suppression of root organogenesis (Fig. 7A). These results suggest that Ca^2+^ signals depend on auxin signaling during root development in leaf explants. The experiments using *glr3.3 glr3.6* double mutants indicated that GLR-mediated Ca^2+^ propagation is not involved in root organogenesis (Fig. 8E). These results are consistent with a previous report that wound-induced expression of *JASMONATE-ZIM-DOMAIN PROTEIN 10* (*JAZ10*) is highly suppressed by *glr3.3 glr3.6* mutations in systemic tissues, but not in local tissues [37]. Therefore, wound-induced local Ca^2+^ signals would affect auxin-dependent root organogenesis in leaf explants. Taken together, we propose a working model for the role of ROS and Ca^2+^ in de novo root organogenesis (Fig. S4). Wound-induced ROS and Ca^2+^ signals are both required for root development, whereas ROS are only partially dependent on auxin signaling.

Our findings demonstrate that inhibition of RBOH activity by DPI treatment largely suppresses de novo root organogenesis (Fig. 2E). However, specific *rboh* mutants exhibited different phenotypes. The rooting rates of *rbohD*, *rbohF*, and *rbohDF* mutants were higher than that of Col-0 (Fig. 8B-D), suggesting that RBOHD and RBOHF negatively regulate root development in leaf explants. These two contrary results suggest that RBOH-produced ROS are required for root organogenesis, while individual RBOHs serve different functions. Enhanced root development in *rbohD* mutants may be due to the accelerated accumulation of JA after wounding, which promotes root regeneration in leaf explants [5, 12]. However, the degree of ROS production could be important for root development. The inhibition of ROS production by treatment with a general RBOH inhibitor, DPI, can be harmful; however, the inhibition of a specific RBOH by genetic mutation can be beneficial for root development. In support of this idea, previous reports have demonstrated that treatment with exogenous hydrogen peroxide promotes adventitious root development; however, increase in the concentration and treatment period suppress root development [13, 38]. Experiments using multiple *rboh* mutants should be conducted to elucidate the relationship between the degree of ROS production and root development.

The role of RBOH-produced ROS in root development in intact plants has been widely studied. RBOHD and RBOHF mediate ABA- and hydrogen sulfide-induced inhibition of primary root growth through the suppression of auxin responses [18, 39]. In contrast, the production of ROS by RBOHs triggers cell wall remodeling and facilitates lateral root emergence [19]. RBOHC functions are important in root hair development [20]. The functions of RBOH even differ between hypocotyl and leaf explants. While RBOHD positively regulates adventitious root development in hypocotyl explants [13], it negatively affects leaf explants (Fig. 8B). Therefore, RBOH activities may be regulated differently regulated in different tissues for the precise regulation of root development. Further studies on the role of each RBOH family in different types of tissues would provide detailed tissue-specific molecular mechanisms of ROS-mediated root development.

Auxin is important for de novo root organogenesis in explants [4, 7]; therefore, previous studies have mainly focused on auxin signaling pathways. Our findings demonstrate that inhibition of ROS and Ca^2+^ signals suppresses the rooting rates of leaf explants, but does not affect the expression of auxin-related genes (Figs. 3 and 6). As ROS modulate adventitious root formation by regulating the expression of genes involved in auxin transport and biosynthesis in hypocotyl explants [13], the ROS-auxin relationship may vary between different tissues.

The suppression of Ca^2+^ signals by EGTA treatment inhibited root development; however, auxin treatment restored these negative effects (Fig. 7A). These data suggest that Ca^2+^ signals depend on auxin signaling. As the inhibition of Ca^2+^ signals did not affect the expression of auxin-related genes and auxin responses (Figs. 6 and 7B), Ca^2+^ signals may act downstream of the auxin signaling pathways during root development in leaf explants. In support of this, CMI1 links Ca^2+^ signals to auxin signaling pathways and affects root development [22]. Additionally, the Ca^2+^-dependent protein kinase is activated by auxin, which is an important process for adventitious root formation in cucumber [24]. In combination with our data, Ca^2+^ signals could activate proteins that mediate auxin-induced root development in leaf explants. Identification of the detailed molecular mechanisms of the Ca^2+^-auxin relationship will be beneficial in understanding how de novo root organogenesis occurs in leaf explants.

Our data showed that detachment of leaves produced ROS not only in the wound site but also in the distant part from the wound site (Fig. 1). Previous reports have demonstrated that wounding induces ROS and Ca^2+^ waves, thus accumulation of ROS and Ca^2+^ is observed in the distal tissues within several minutes after wounding [10, 12]. Similar to these reports, wounding in the detached leaves might produce ROS waves, thus ROS signals were observed in the total leaf area (Fig. 1). Systemic accumulation of ROS would be important for root organogenesis, because inhibition of the systemic ROS by treatment of DPI largely suppressed root organogenesis (Fig. 2). Because ROS influence phytohormone signals and expression of genes having diverse functions [12], ROS-activated signals in the distant part of the leaves might send long-distance signals back to the wound site for regulation of root organogenesis.

## Methods

### Plants materials and growth conditions

*Arabidopsis thaliana* ecotype Columbia (Col-0) was obtained from Dr. Chung-Mo Park’s lab at the Seoul National University and it was originally from the Arabidopsis Biological Resource Center (ABRC, Ohio State University, Columbus, OH, USA). The *rbohC* (N2259), *rbohD* (N9555)*, rbohF* (N9557)*, rbohDF* (N9558)*, glr3.3* (N663463)*,* and *glr3.6* (N663316) mutants were obtained from the Nottingham Arabidopsis Stock Centre (NASC, Nottingham, UK). The *DR5*_*rev*_*:GFP* (CS9361) was obtained from the ABRC. The *glr3.3 glr3.6* double mutants were obtained from crossbreeding *glr3.3* and *glr3.6* mutants. S.Y.S. and H.J.L. performed identification of all plant materials. Newly generated plant materials in this study are not deposited in a publicly available herbarium, but they are available from the corresponding author on reasonable request. Seeds were surface-sterilized in 75% (v/v) ethanol with 0.03% (v/v) Triton X-100 and then washed twice using 70% (v/v) ethanol before stratification at 4 °C. After three days, the seeds were transferred to a growth room set at 24 °C with 40–50% humidity under long-day conditions (16 h-light, 8 h-dark). The seedlings were grown on 1/2 X Murashige and Skoog-agar (MS-agar) plates containing 0.7% (w/v) of plant agar with pH 5.7. White light with an intensity of 100 μmol m^−2^ s^−1^ was applied using fluorescent FL40EX-D tubes (Focus, Bucheon, Korea).

To generate *WOX11*_*pro*_*:GUS* transgenic plants, 4.87-kb upstream sequences of the *WOX11* gene were amplified using pWOX11-F (5’-AATGGATCCCTAACTGTTACGATTGAATTCAAACGATA-3’) and pWOX11-R (5’-TTGGCGCGCCTGCTTTGAAGAATATTGATATTA-TCTGGTG-3’). The PCR products were combined into a modified pBA-GUSII vector using BamHI and AscI restriction enzymes.

### Induction of de novo root organogenesis using leaf explants

Seedlings were grown for 9 days and first rosette leaves were detached. The explants were incubated on Gamborg B5 basal medium-agar (B5-agar) plates containing 0.7% (w/v) of plant agar with pH 5.7 for up to 14 days to induce de novo root organogenesis. For DPI treatment, explants were incubated on B5-agar plates containing 1 or 10 μM of DPI. For EGTA treatment, explants were incubated on B5-agar plates containing 0.5, 1, or 1.5 mM of EGTA. For NAA treatment, explants were incubated on B5-agar plates containing 0.1 μM of NAA.

### ROS staining

Seedlings were grown on MS-agar plates for 9 days and first rosette leaves were incubated on B5-agar plates containing 1μM of DPI or 0.5 mM of EGTA for the indicated time periods in figures. For DAB staining, explants were immersed in DAB staining solution containing 40 mg of 3,3'-diaminobenzidine and 20 μL of Tween 20 in 40 mL of distilled water, and then vacuum-infiltrated for 5 min. The seedlings were then incubated for approximately 8 h in the darkness at 24 °C without shaking. After the staining, DAB solutions were removed and then explants were immersed in 70% (v/v) ethanol to remove the chlorophyll. For NBT staining, explants were immersed in NBT staining solution containing 70 mg of nitroblue tetrazolium and 13 mg of sodium azide in 20 mL of 10 mM potassium phosphate buffer (pH 7.8) and vacuum-infiltrated for 2 min. Explants were then incubated for 2 h in the darkness at 24 °C without shaking. The NBT staining solution was then removed and the plant materials were immersed in 70% (v/v) ethanol to remove the chlorophyll. The plant materials were mounted on slide glasses and photographed using a Nikon D5600 digital camera. The ImageJ software was used to measure the DAB and NBT signal intensity.

### GUS staining

The *pWOX11:GUS* transgenic plants were grown for 9 days on MS-agar plates and first rosette leaves were incubated on B5-agar plates containing 1 μM of DPI or 0.5 mM of EGTA for the indicated time periods in figures. Explants were fixed with 90% cold acetone for 20 min on ice. Explants were then washed two times using rinsing solution containing 50 mM of sodium phosphate pH 7.2, 0.5 mM of K_3_Fe(CN)_6_, and 0.5 mM of K_4_Fe(CN)_6_. Washed leaves were completely submerged in staining solution containing 2 mM of X-Gluc (Duchefa) in the rinsing solution and incubated at 37 °C for 16 h. Explants were then incubated in 70% ethanol for 24 h to remove the chlorophyll. Stained leaves were mounted on slide glasses and photographed using Nikon D750 digital camera. The ImageJ software was used to measure the GUS signal intensity.

### Fluorescence imaging

The *DR5*_*rev*_*:GFP* transgenic plants were grown for 9 days on MS-agar plates. First rosette leaves were detached and incubated on B5-agar plates containing 1 μM of DPI or 0.5 mM of EGTA for the indicated time periods in figures. The plant materials were mounted on the slide glasses and abaxial side of the leaves were observed. GFP and chlorophyll fluorescence imaging were performed using an LSM 800 confocal microscope (Carl Zeiss). The excitation and emission wavelengths of GFP fluorescence were 488 and 500–600 nm, respectively. For analysing chlorophyll fluorescence, excitation and emission wavelengths were 561 and 560–700 nm, respectively. Fluorescence images were analyzed using the ZEN 2.5 LITE software. To measure fluorescence intensity of GFP signals, the ImageJ software was used. Three to five biological repeats were analyzed.

### RNA extraction and gene expression analysis by quantitative PCR

Plants were grown for 9 days on MS-agar plates. First rosette leaves were detached and incubated on B5-agar plates containing 1 μM of DPI or 0.5 mM of EGTA for the indicated time periods in figures. Total RNA was extracted from the explants using the Trizol (Thermo Fisher Scientific) or the RNeasy Plant Mini Kit (QIAGEN, Hilden, Germany) according to the manufacturer’s recommendations. First-strand complementary DNA synthesis was performed using AccuPower CycleScript RT PreMix (Bioneer) according to the manufacturer’s protocol. QPCR was performed using TOPreal qPCR 2X PreMIX (SYBR Green with low ROX, Enzynomics). Primers used for qPCR were listed in Table S1. The gene expression was normalized using *UBQ10* as a reference gene.

### Statistical analysis

All statistical methods as well as the number of biological replicates in each assay are annotated in the figure legends. To determine statistically significant differences, one-way analysis of variance (ANOVA) with post hoc Tukey’s test and Student’s *t*-test were performed using Rstudio and Excel software, respectively.

## Supplementary Information

**Additional file 1: Fig. S1.** Effects of DPI on root organogenesis in the leaf explants. **Fig. S2.** Observation of auxin responses using *DR5*_*rev*_*:GFP* at 0 DAC. **Fig. S3.** Effects of EGTA on root organogenesis in the leaf explants of *rboh* mutants. **Fig. S4.** Proposed working model for the role of ROS and Ca^2+^ on root organogenesis. **Table S1.** Primers used in this study.

## Data Availability

All generated or analysed data were included in this article. The raw datasets obtained during the current study are available from the corresponding author on reasonable request.

## Abbreviations

ABA: Abscisic acid
AFB1: AUXIN SIGNALING F BOX PROTEIN 1
ASA1: ANTHRANILATE SYNTHASE α1
Ca^2^^+^: Calcium ion
CMI1: Ca^2^^+^-DEPENDENT MODULATOR OF ICR1
DAB: 3,3'-Diaminobenzidine
DAC: Days after culture
DPI: Diphenylene iodonium
EGTA: Ethylene glycol tetraacetic acid
ERF109: ETHYLENE RESPONSE FACTOR 109
GFP: Green fluorescent protein
GLR: GLUTAMATE RECEPTOR-LIKE
GUS: β-Glucuronidase
ICR1: INTERACTOR OF CONSTITUTIVELY ACTIVE ROP1
JA: Jasmonic acid
JAZ10: JASMONATE-ZIM-DOMAIN PROTEIN 10
LBD: LATERAL ORGAN BOUNDARIES DOMAIN
NAA: Naphthalene-1-acetic acid
NAC1: NAC DOMAIN CONTAINING PROTEIN 1
NBT: Nitroblue tetrazolium
PIN: PIN-FORMED
RBOH: RESPIRATORY BURST OXIDASE HOMOLOG
ROS: Reactive oxygen species
RT-qPCR: Reverse transcription-quantitative polymerase chain reaction
TIR1: TRANSPORT INHIBITOR RESPONSE 1
WOX: WUSCHEL-RELATED HOMEOBOX
YUC: YUCCA
