# Correction: Wound-induced signals regulate root organogenesis in Arabidopsis explants

**DOI:** 10.1186/s12870-023-04291-y

**Published:** 2023-05-22

**Authors:** Seung Yong Shin, Su-Jin Park, Hyun-Soon Kim, Jae-Heung Jeon, Hyo-Jun Lee

**Affiliations:** 1 Plant Systems Engineering Research Center, Korea Research Institute of Bioscience and Biotechnology, Daejeon, 34141 Korea; 2 Department of Functional Genomics, KRIBB School of Bioscience, University of Science and Technology, Daejeon, 34113 Korea; 3 Department of Biosystems and Bioengineering, KRIBB School of Biotechnology, University of Science and Technology, Daejeon, 34113 Korea

**Correction:** ***BMC Plant Biol*** **22, 133 (2022)**

10.1186/s12870-022-03524-w

Following publication of the original article [1], the authors identified an error in some of the figure captions. Unconverted character was found in the following captions in **boldface**:

- Figure 2 ‘B5-agar plates containing 1 M DPI’ should be ‘B5-agar plates containing 1 **µ**M DPI’
- Figure 3 ‘B5-agar plates containing 1 M DPI’ should be ‘B5-agar plates containing 1 **µ**M DPI’
- Figure 4 ‘B5-agar plates containing 1 M DPI with or without 0.1 M NAA’ should be ‘‘B5-agar plates containing 1 **µ**M DPI with or without 0.1 **µ**M NAA’
- Figure 4 ‘B5-agar plates containing 1 M DPI’ should be ‘B5-agar plates containing 1 **µ**M DPI’
- Figure 7 ‘EGTA with or without 0.1 M NAA’ should be ‘EGTA with or without 0.1 **µ**M NAA’

The correction do not affect the Conclusions of the article. The original article [1] has been corrected.
