# Supplementary material for: Wound-induced signals regulate root organogenesis in Arabidopsis explants
Source: BMC Plant Biol. 2022 Mar 22;22:133. doi: 10.1186/s12870-022-03524-w (PMC8939181; doi:10.1186/s12870-022-03524-w)
Supplement: Supplementary file 1 — Additional file 1: Fig. S1. Effects of DPI on root organogenesis in the leaf explants. Fig. S2. Observation of auxin responses using DR5rev:GFP at 0 DAC. Fig. S3. Effects of EGTA on root organogenesis in the leaf explants of rboh mutants. Fig. S4. Proposed working model for the role of ROS and Ca2+ on root organogenesis. Table S1. Primers used in this study. [file 12870_2022_3524_MOESM1_ESM.pdf]

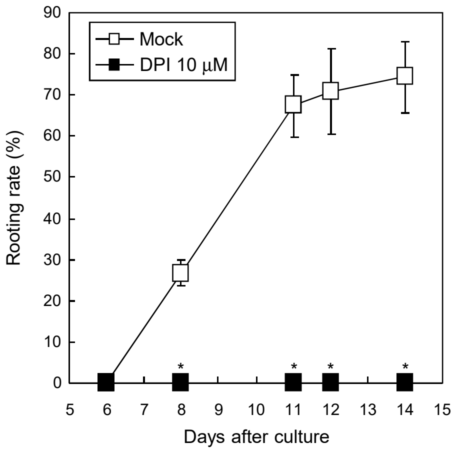


**Fig. S1** Effects of DPI on root organogenesis in the leaf explants.
Leaf explants from the 9-day-old Col-0 seedlings grown on Murashige and Skoog (MS)-agar plates under long-day conditions (16 h light/8 h dark) were incubated on B5-agar plates containing 10 μM DPI up to 14 d. Rooting rates of three biological replicates were averaged and statistically analyzed using Student's *t*-test (*, $P < 0.05$; difference from Mock). Each replicate contains 17-22 explants. Whiskers indicate ± standard deviations (SD).

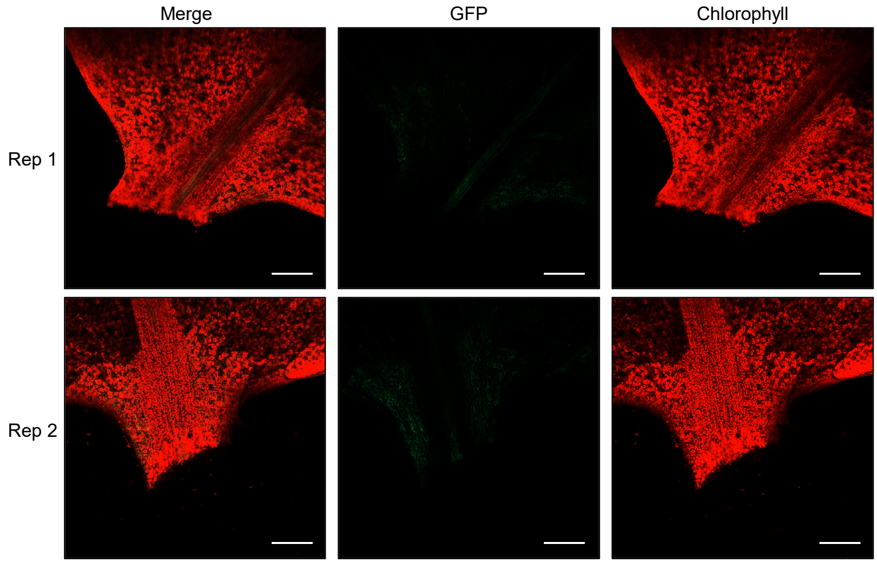


**Fig. S2** Observation of auxin responses using *DR5*$_{rev}$*:GFP* at 0 DAC.
Leaf explants from the 9-day-old *DR5*$_{rev}$*:GFP* transgenic seedlings grown on MS-agar plates under long-day conditions were used for confocal microscopy. Two represent replicates were displayed. Fluorescence signals near the wound site of the explants were analyzed. DAC, days after culture. Scale bars indicate 0.2 mm.

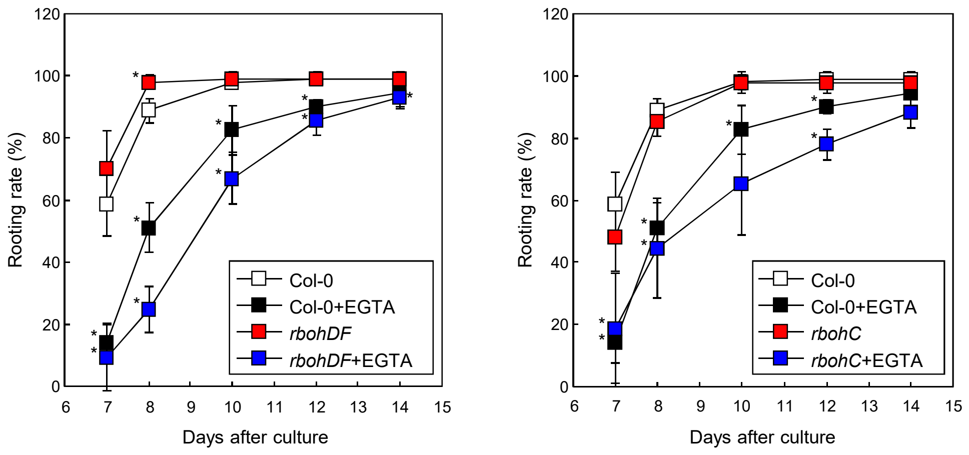


**Fig. S3** Effects of EGTA on root organogenesis in the leaf explants of *rboh* mutants.
Leaf explants from the 9-day-old Col-0, *rbohC* and *rbohDF* seedlings were incubated on B5-agar plates containing 0.5 mM EGTA for up to 14 d. Biological triplicates were averaged and statistically analyzed using Student's *t*-test (*, $P < 0.05$; difference from Col-0). Each replicate contains 16-25 explants. Whiskers indicate ± SD.

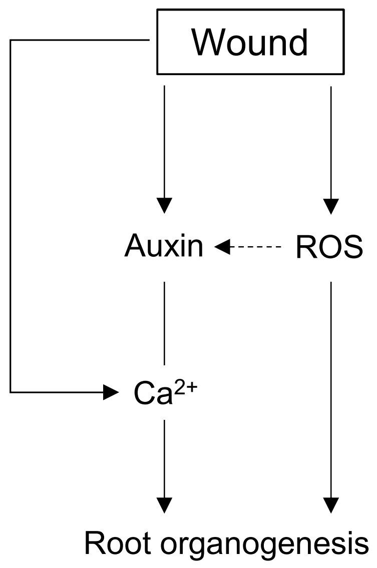


**Fig. S4** Proposed working model for the role of ROS and $Ca^{2+}$ on root organogenesis.
Wound-induced reactive oxygen species (ROS) and $Ca^{2+}$ signals are required for root organogenesis. Auxin and ROS signals have some crosstalk, and auxin might act downstream of ROS signaling. $Ca^{2+}$ signals are necessary for auxin-induced root organogenesis from the explants.

| Primers | Sequences | Usage |
|---|---|---|
| UBQ10-F | CGACCCTTCACTTGGTGTTG | RT-qPCR |
| UBQ10-R | TTTCCAGCGAAGATGAGACG | 〃 |
| YUC2-F | CGGTTAGGGTTAGTTCGACC | 〃 |
| YUC2-R | GAACCTCAATATCCTCAGCG | 〃 |
| YUC4-F | GGAACGGGGCAAAGTTTCTG | 〃 |
| YUC4-R | CTCGTGAAACCCACCGTGTA | 〃 |
| NAC1-F | CATCCTCCCAATCATTCTCTG | 〃 |
| NAC1-R | GAATGAGTCGAGGCCTGTGA | 〃 |
| WOX11-F | AAGTGACGAGAGGAGGAATA | 〃 |
| WOX11-R | ATGTCTGTCTTGGAACCAG | 〃 |
| TIR1-F | GAATTGGGCACTGGTGGGTA | 〃 |
| TIR1-R | AGCCGACTGCAAACCGAATA | 〃 |
| AFB1-F | TACGCTACTGTCCGAATGCC | 〃 |
| AFB1-R | CACCCTCAGTTCTCGCAGTT | 〃 |
| PIN1-F | TGGTAATTTCTCCGGCGACC | 〃 |
| PIN1-R | GTGTCTGGAAACTGCTCGGA | 〃 |
| PIN2-F | TTCTTTGGCAGGCGTTTAGC | 〃 |
| PIN2-R | TGAAGCACCACGATCTGCAC | 〃 |
| PIN3-F | ACGTGGCAATGCCCAAAATC | 〃 |
| PIN3-R | GGCAAACGTTGCCACTGAAT | 〃 |
| PIN7-F | AAATGGTGAAAACAAAGCTGGT | 〃 |
| PIN7-R | GGGTTTAGCTCTGCTGTGGA | 〃 |

**Table S1** Primers used in this study.

The primers used were designed using the Primer Blast (https://www.ncbi.nlm.nih.gov/tools/primer-blast). F, forward primer; R, reverse primer.
